# Assessment of utilization pattern of fixed dose drug combinations in primary, secondary and tertiary healthcare centers in Nepal: a cross-sectional study

**DOI:** 10.1186/s40360-017-0176-z

**Published:** 2017-11-02

**Authors:** Arjun Poudel, Mohamed Izham Mohamed Ibrahim, Pranaya Mishra, Subish Palaian

**Affiliations:** 1 Research Associate, School of Clinical Sciences, Faculty of Health, Queensland University of Technology, Brisbane, Australia; 2 Department of Hospital and Clinical Pharmacy, Manipal Teaching Hospital, Phulbari-11, Pokhara, Nepal; 3 Clinical Pharmacy and Practice Section, College of Pharmacy, Qatar University, Doha, Qatar; 4 Department of Pharmacology, American University of the Caribbean School of Medicine, University Drive at Jordan Road, Cupecoy, St. Maarten, Coral Gables, USA; 5 Department of Pharmacy Practice, College of Pharmacy, Gulf Medical University, Ajman, United Arab Emirates

**Keywords:** Drug utilization, Fixed dose drug combinations, Nepal, Prescribing pattern, Healthcare centers

## Abstract

**Background:**

Prescription practices, especially in South Asian countries, have come under investigation for quality. Although there have been no studies in Nepal that have analyzed the prescription pattern of FDCs for different levels of health care centers, several studies from Nepal and other countries in the region have revealed poor medicine use practices, including irrational use of fixed-dose drug combinations (FDCs). This research aimed at assessing the utilization pattern of FDCs among primary (PHC), secondary (SHC) and tertiary health care (THC) centers in Western region of Nepal.

**Methods:**

A cross-sectional descriptive study was conducted at primary, secondary and tertiary health care centers in Western Nepal. One hundred prescriptions from each health care center were chosen through systematic random sampling. The International Network for Rational Use of Drug (INRUD) indicators were used to assess the rationality of prescribing. Both descriptive and inferential statistics were applied. The alpha level used was 0.05.

**Results:**

At the PHC center, 206 medicines were prescribed, of which 20.0% were FDCs. Antimicrobials were the most prescribed FDCs (57.1%). The unit prices of all FDCs were below 100 Nepalese Price Rupees (NPRs). At the SHC center, 309 medicines were prescribed, and 30% were FDCs. Vitamins, minerals and dietary supplements were the most prescribed FDCs (25.8%). The costs of 63.5% of FDCs were below 100 NPRs. At the THC center, 33.5% of 270 medicines were FDCs. As at the SHC center, vitamins, minerals and dietary supplements were the most prescribed FDCs (40.6%). The costs of 50.5% of FDCs were below 100 NPRs.

**Conclusions:**

FDCs were used extensively at different health care centers. The number of prescription in private centers, following established guidelines and the essential drug list (EDL), was much lower. The cost associated with the utilization of FDCs was higher in private sectors compared to public health care centers. In certain cases, the use of FDCs was questionable, and this study found a low use of essential medicines. Education to improve prescription practices at different healthcare levels is recommended.

**Electronic supplementary material:**

The online version of this article (10.1186/s40360-017-0176-z) contains supplementary material, which is available to authorized users.

## Background

Medicine use studies are conducted to monitor, assess and change the prescribing habits of practitioners. The main purpose of these studies is to make health care more cost-effective and rational [1]. Drug utilization has been defined as “the marketing, distribution, prescription and use of medicines in a society, with special emphasis on the resulting medical, social and economic consequences” [2]. Periodic evaluations of drug utilization patterns are necessary to increase the therapeutic benefits of medicines, decrease adverse effects and, ultimately, modify the prescription of medicines as needed.

Rational prescription practices mean “making a diagnosis, estimating prognosis, obtaining the best possible effect with the least number of medicines in the shortest period and at reasonable cost and monitoring the effects of the treatment” [3]. In both developed and developing countries, the irrational and inappropriate use of medicines is a major concern [4]. The prescribing behavior of a practitioner depends on several factors, including commercial publicity, government regulations, academic literature, patient behaviors, and influences from pharmaceutical companies. Inappropriate responses to these factors lead to prescription errors and irrational prescribing habits, both of which are very common in clinical practice.

In a low-income country like Nepal, high numbers of people lack access to medicine due to its poor availability and restrictive cost. Though 42% of the health budget is spent on medicines, the availability of medicines in health posts (HPs) and clinics is inconsistent, contributing to the higher prevalence of the inappropriate drug use [5] such as the irrational fixed dose drug combinations (FDCs) that are readily available in the market. In many instances, the use of irrational FDCs is not justified. FDCs are medicines that contain two or more active components in fixed proportions in a single dosage form [6]. Rational combinations improves quality of life of many people and can be of great benefit to the healthcare system [7] such as the antitubercular medicines, antiretroviral medicines and some antihypertensive medicines. Irrational combinations, on the other hand are equally harmful such as the combination of nonsteroidal anti-inflammatory drugs (NSAIDs) with muscle relaxants, NSAIDs with H2 blockers, cough syrups with two or more antihistamines, steroids, antibiotics and bronchodilators [8]. Irrational FDCs have a combination of medicines that may be used to treat a disorder without achieving a correct diagnosis [4]. The FDC may not contain the required quantity of each individual drug, and the combination may not be synergistic. Health care centers in Nepal are flooded with irrational FDCs, while the patients are deprived of essential medicines [9].

The prevalence of irrational prescription practices, especially in South Asian countries, has led to special investigations into how and why medicines are prescribed.

Several interventions have however, been initiated such as the banning of irrational combinations, withdrawing them from the market and ceasing the manufacturing of irrational FDCs [10, 11]. Despite these interventions, irrational FDCs are still available in the market and are used extensively. The interventions are not successful due to several reasons. One such reason relates to exporting of FDCs that are banned in a particular country to the neighboring countries [12]. Such exports hinder the interventional process of government or individuals trying to weed out irrational combinations from their country. Studies from Nepal and other South Asian countries concluded that the quality of prescription is poor, with the common overuse of antimicrobials and the irrational use of FDCs, particularly in private sectors [13–15]. In Nepal, the irrational and inefficient use of medicines is prevalent at all levels of health care [16]. Prescribing irrational FDCs is the rule rather than the exception, with up to seven items being prescribed for a single disease [13]. Even with the frequent use of FDCs, finding the optimal dose and optimal combination is still a matter of trial and error. The reason many FDCs are prescribed appears to be irrational [17], though the practice is common at various health care centers. A study at a tertiary care teaching hospital in Nepal found FDCs accounted for 47% of the total medicines prescribed, even though their use was inappropriate in numerous cases [18]. Irrational FDCs can also lead to adverse drug reactions (ADRs). A study involving a community pharmacist from western Nepal reported that the FDC of ibuprofen and paracetamol accounted for the highest incidence of ADRs [19].

In Nepal, there have been no studies that analyzed the prescription pattern of FDCs at different levels of health care centers. This study was the first to analyze utilization patterns of FDCs in private and public sectors. In Nepal, primary health care (PHC) centers are managed by the government. The secondary health care (SHC) centers and tertiary health care (THC) centers in our study were privately managed. Our study examined the prevalence of FDCs and the cost associated with their use; we can corroborate past reports of widespread irrational FDC use in the private sector [20]. Our findings may also help concerned regulatory bodies analyze FDCs used in different health care centers to mitigate the free flow of irrational FDCs. The study can also create awareness among all health care professionals regarding the hazards associated with the irrational use of FDCs.

### Study objectives

The study was designed to accomplish the following:

1. To analyze the prescription pattern of FDCs in primary, secondary and tertiary health care centers,

2. To categorize the FDCs prescribed from the primary, secondary and tertiary health care centers based on their therapeutic class, and.

3. To analyze the cost associated with the FDCs prescribed from the primary, secondary and tertiary health care centers.

## Methods

### Study type

A cross-sectional descriptive study was conducted from February to May 2009, analyzing the utilization pattern of FDCs in selected primary, secondary and tertiary health care centers in Western Nepal.

### Study site

The study was carried out in selected primary, secondary and tertiary health care centers in Pokhara, Western regional of Nepal. The details of each center are mentioned below.

### Primary health care (PHC) center

The Nepalese PHC system operates at different levels: PHC centers, health posts (HP) and the sub-health posts (SHP). For the majority of the rural population, the PHC center serves as the first level of contact with the healthcare system. In this study, the HP at the Batulechaur Village Development Committee (VDC) of Kaski district was selected as a PHC center. This health care center is located approximately 10 km from Pokhara city, with an average patient flow of 20 patients per day. This health care center is regulated by the government, and it is the government which caters to the primary health care needs of Batulechaur VDC. Minor diseases like acute respiratory infections, wounds, dental caries, skin diseases and worm infestation are managed in these health care centers.

### Secondary health care (SHC) center

Charak Hospital, a private hospital in Pokhara, was selected as a SHC center. It has an average bed occupancy of approximately 100 patients and an average outpatient count of approximately 200 patients per day. The hospital consists of various specialty departments (both clinical and non-clinical).

### Tertiary health care (THC) center

Manipal Teaching Hospital (MTH), a private institution, was selected as a THC center for our study. MTH is a 700-bed tertiary care teaching hospital with average bed occupancy of approximately 200 patients and an average outpatient rate of approximately 400 per day. The hospital consists of various specialty departments (both clinical and non-clinical). The hospital caters to the healthcare needs of patients of Western and Midwestern region of Nepal.

### Population and sampling procedure

The target population of this study is patients visiting pharmacies of each health care setting with a prescription from outpatient departments. Patients’ verbal informed consent was obtained, and patients who were not willing to participate in the study, as well as inpatients from each health care center were excluded from the study.

Based on the time and phase of the study, sample size was estimated for 100 patients per setting, leading to a total of 300 patients. Systematic random sampling was used to select patients with prescriptions from each health care setting. Five patients were sampled every alternate day in PHC, SHC, and THC centers for a period of three months until 100 prescriptions were encountered.

### Study tools

The International Network for Rational Use of Drug (INRUD) encounter form was modified and used for data collection (Additional file 1). The cost per dispensed quantity was not included in the original INRUD encounter form. Thus, the original form was modified and divided into a prescribing indicator, a patient care indicator and a cost indicator (USD 1 = NPR 75). In addition, the National model list of essential medicines for Nepal, the WHO Model List of Essential Medicines, and the Nepalese National Formulary (NNF) were used as standard resources to check the prescription pattern and the medicines listed in these sources were considered rational.

### Data collection procedure

Patients visiting pharmacies with a prescription from each health care setting were enrolled in the study. The prescriptions from the patients were used as a source of data. The patients were interviewed after the medicines had been dispensed to them for their knowledge regarding dose, duration, and frequency of medicines. The information gathered was noted on the data collection form (Additional file 1).

### Data analysis procedure

The completed encounter forms were analyzed per the study objectives. Parameters such as the “details of medicines present in different formularies and drug list,” the “therapeutic category of medicines prescribed” and the “cost analysis for the medicines prescribed” were analyzed. The data obtained were entered in a Microsoft Excel spreadsheet and analyzed. Statistical Package for the Social Sciences (SPSS) version 16.0 (SPSS Inc. Released 2007Chicago, SPSS, Inc.) was used to carry out the descriptive and inferential statistics. A Kolmogorov-Smirnov test was used to check the normality of the data. A Kruskal-Wallis test was used to compare the difference between three health care centers based on cost. A *p* value of less than 0.05 was considered statistically significant. Post-hoc analysis was carried out using Mann-Whitney U test between two groups to determine any differences. A Bonferroni adjustment using the formula k(k-1)/2, where k is the number of groups or conditions, was performed to remove the type I error. After applying the formula, we found that any pair of data has to achieve a significant value on a paired test smaller than 0.05/3 = 0.017 to be significant at the 0.05 level.

### Pilot study

A pilot study was carried out in selected primary, secondary and tertiary health care centers in Western Nepal [20]. Twenty-five, fifty and seventy-five prescriptions from each primary, secondary and tertiary health care center, respectively, were collected. The data collection form was improved by including the parameters assessing the patients’ knowledge on medicines regarding dose, duration and frequency. The pilot study also provided insights on feasibility of conducting the main research.

## Results

### Part I: Primary health care center

Altogether, 100 prescriptions were encountered from the PHC center. The median (IQR) age of the patients was 36 (18–60) years, and majority were female (64%). A total of 206 medicines were prescribed, of which 20.4% (*n* = 42) were FDCs. Analysis of the prescriptions was further carried out based on the study objectives.

#### Details of medicines present in different formularies and drug lists

**Fig. 1:**
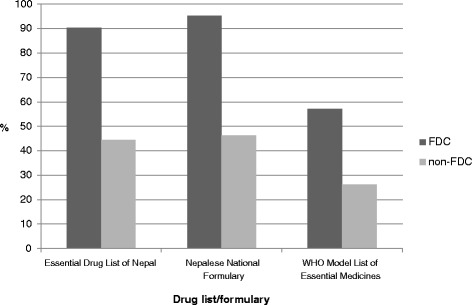
Number of drugs present in different formularies and drug lists in PHC center

Out of the total 42 FDCs prescribed, 90.4% (*n* = 38) were from the Essential Drug List (EDL) of Nepal (3rd Revision 2002), 95.2% (*n* = 40) were from the NNF, and 57.1% (*n* = 24) were from the World Health Organization (WHO) Model List of Essential Medicines (15th Edition). The details are shown in Fig. 1.

#### Therapeutic category of medicines prescribed

**Fig. 2:**
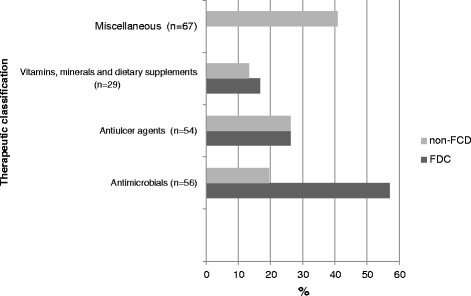
Therapeutic category of drugs prescribed in PHC center

Among the 206 medicines prescribed, antimicrobials made up to 27.1% (*n* = 56), and antiulcer medicines accounted for 26.2% (*n* = 54). Among the 42 FDCs prescribed, 57.1% (n = 24) were antimicrobials, and 26.1% (*n* = 11) were antiulcer medication. The details are shown in Fig. 2.

#### Cost analysis for the medicines prescribed

Out of 206 medicines prescribed, 85.4% (*n* = 176) of them cost less than 100 Nepalese price rupees (NPRs), while 30 medicines were between 101 and 200 NPRs. The cost of all the 42 FDCs prescribed was below 100 NPRs.

### Part II: Secondary health care center

A total of 100 prescriptions were derived from the SHC center. The median (IQR) age of the patients was 33 (28–45) years, and majority were female (60%). A total of 309 medicines were prescribed, of which 30% (*n* = 93) were FDCs. The prescription analysis was further carried out based on the study objectives.

#### Details of medicines present in different formulations and drug lists

**Fig. 3:**
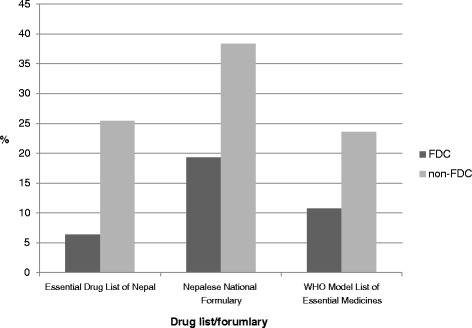
Number of drugs present in different formularies and drug lists in SHC center

Of the 93 FDCs prescribed, 6.4% (*n* = 6) were from the EDL of Nepal (3rd Revision 2002), 19.3% (*n* = 18) were from the NNF, and 10.7% (*n* = 10) were from the WHO Model List of Essential Medicines (15th Edition). The details are shown in Fig. 3.

#### Therapeutic category of medicines prescribed

**Fig. 4:**
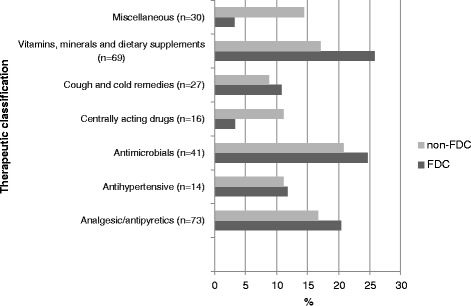
Therapeutic category of drugs prescribed in SHC center

Among the 309 medicines prescribed, vitamins, minerals and dietary supplements were commonly prescribed, accounting for 19.7% (*n* = 61) of the medicines. Antimicrobials were even more prevalent at 22% (*n* = 68). Similarly, among the 93 FDCs, the most prescribed medicines were vitamins, minerals and dietary supplements at 25.8% (*n* = 24), followed by antimicrobials at 24.7% (*n* = 23). The details are shown in Fig. 4.

#### Cost analysis for the medicines prescribed

**Fig. 5:**
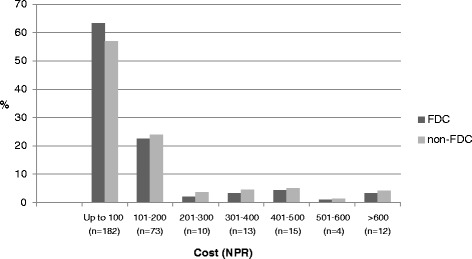
Cost analysis for the drugs prescribed in SHC center

Among the 309 medicines prescribed, 58.9% (*n* = 182) cost less than 100 NPRs, followed by 23.6% (*n* = 73) with costs in the range of 101–200 NPRs. Among the 93 FDCs, 63.4% (*n* = 59) were less 100 NPRs, and 22.5% (*n* = 21) of the medicines were in the price range of 101–200 NPRs. The details are shown in Fig. 5.

### Part III: Tertiary health care center

Altogether, 100 prescriptions were encountered from the THC center. The median (IQR) age of the patients was 32 (19–47) years, and majority were female (66%). A total of 270 medicines were prescribed, of which 33.5% (*n* = 91) were FDCs. Further analysis was carried out based on the study objectives.

#### Details of medicines present in different formularies and drug lists

**Fig. 6:**
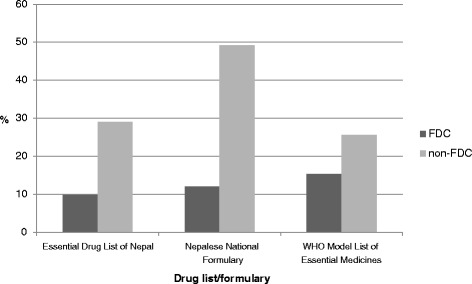
Number of drugs present in different formularies and drug lists in THC center

Out of the total 91 FDCs prescribed, 9.8% (n = 9) were from the EDL of Nepal (3rd Revision 2002), 12.0% (*n* = 11) were from the NNF, and 15.3% (*n* = 14) were from the WHO Model List of Essential Medicines (15th Edition). The details are shown in Fig. 6.

#### Therapeutic category of medicines prescribed

**Fig. 7:**
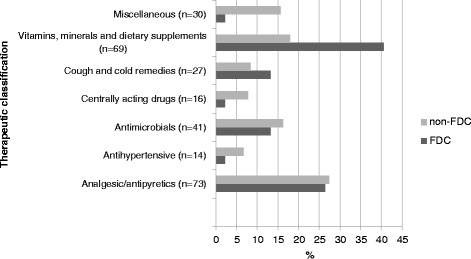
Therapeutic category of drugs prescribed in THC center

Among the 270 medicines prescribed, analgesic/antipyretics were the most prescribed medicines at 27% (*n* = 73), followed by vitamins, minerals and dietary supplements, which accounted for 25.5% (*n* = 69). Similarly, among the 91 FDCs, the most prescribed medicines were vitamins, minerals and dietary supplements with 40.6% (*n* = 37) as FDCs, followed by 26.3% (*n* = 24) that were analgesic/ antipyretics. The details are shown in Fig. 7.

#### Cost analysis for the medicines prescribed

**Fig. 8:**
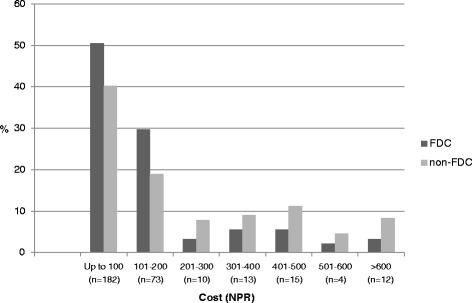
Cost analysis of drugs prescribed in THC center

Among the total 270 medicines prescribed, 43.7% (*n* = 118) cost less than 100 NPRs, and 22.5% (*n* = 61) were in the range of 101–200 NPRs. Among the 91 FDCs, 50.5% (*n* = 46) had prices below 100 NPRs, followed by 29.6% (*n* = 27) that were in the range of 101–200 NPRs. The details are shown in Fig. 8.

#### Difference in the use of fixed dose drug combinations among primary, secondary and tertiary health care centers

The difference in the use and cost of FDCs in primary, secondary and tertiary health care centers was compared. A statistically significant difference was found in the utilization pattern of FDCs for the primary, secondary and tertiary health care centers (*p* = 0.000). The details are shown in Table 1.

**Table 1:** Difference of cost (NPR) of FDCs between PHC, SHC and THC centers (*n* = 226)

Health care centers	Median (IQR)	*P* value
Primary Health Care center (*n* = 42)	43 (30–54)	0.000*
Secondary Health Care center (*n* = 93)	84 (45–153)	
Tertiary Health Care center (*n* = 91)	102 (55–180)	

*Kruskal Wallis test at ά = 0.05

A post-hoc analysis using a Mann-Whitney U test among groups showed a statistically significant difference in primary health care centers (p = 0.000) compared to the other groups. A significant difference was observed between primary and secondary health care centers (p = 0.000), as well as between primary and tertiary health care centers (p = 0.000). There was no significant difference between secondary and tertiary health care centers (*p* = 0.998).

## Discussion

This study was the first of its kind to analyze the utilization pattern of FDCs among the three levels (primary, secondary and tertiary) of health care centers in Nepal. The study assessed the prescribing patterns of FDCs in these three levels of health care system based on different formularies and drug lists. The study also analyzed the therapeutic class of the drug prescribed and the cost associated with it.

The PHC centers selected in this study are managed by the government. Due to the immense human cost of disease in Nepal, PHC centers receive the highest allocation in national health spending and about three quarters of the total health budget [21]. PHC centers provide essential health care based on a practical, socially acceptable method made universally accessible to the individuals and community at the lowest possible cost [22]. In our study, out of the 206 medicines prescribed, 20% were FDCs. The average number of medicines per prescription was 2.06. Our findings are slightly lower than studies in PHC centers in plain (also called as Terai) districts that had 2.2 medicines per prescription and centers in hill districts that had 2.1 medicines per prescription [23]. In a similar study in rural districts in Tajikistan the median number of medicines prescribed was 3, ranging from 1 to 8 or more medicines [24].

Approximately 90% of FDCs prescribed were from the EDL of Nepal, 95% were from the NNF, and 57% were from the WHO Model List of Essential Medicines. In the previous study, at the primary healthcare facilities in the Kaski district, the percentage of medicines prescribed from the EDL varied from 70.9% to 74.0% [21]. The higher rate of utilization of FDCs from EDL may be because they were available and free [25] after the government of Nepal decided to offer essential health care services free of charge to all citizens at all health and sub-health posts from mid-January 2008 [26]. Antimicrobials and antiulcer FDCs were prescribed in 57% and 26% of the encounters, respectively. Our observations regarding the prevalence of antimicrobials in the PHC center was similar to that of a study in Ethiopia [27]. All the FDCs prescribed in PHC center were below $1.25.

Secondary health care typically refers to services provided by hospitals. The SHC center selected in the study was a private hospital. During our study period, we found that 30% of the 309 medicines prescribed were FDCs. The average number of medicines per prescriptions was 3.09. In contrast to the finding from the PHC center, we found that only 6.4% of FDCs encountered in SHC center were from the EDL of Nepal, 19.3% were from the NNF, and 10.7% were from the WHO Model List of Essential Medicines. The most widely prescribed FDCs were vitamins, minerals and dietary supplements, which accounted for 24 (25.8%) of the total FDCs, followed by 23 (24.7%) antimicrobials. The WHO has removed vitamin combinations from their list with the comment that vitamins are considered part of nutrition, and combinations should not be used indiscriminately [28]. Among the total FDCs encountered, 63% were less than $1.25, and 22% were in the range of $1.2 - $2.5.

Tertiary health care refers to specialist services mostly provided by the private medical institutions. Similar to the SHC center, the THC center selected for our study was a private hospital. Altogether, 270 medicines were prescribed, out of which 33.5% were FDCs. In Nepal health units, an average of 56% medicines were prescribed by brand names, and FDC formulations contributed up to 46.6% [28]. However, a study from Western Nepal found that FDCs accounted for only 15.8% of medicines prescribed [4]. Similar studies in the same hospital found that FDCs comprised 47% of all prescribed medicines [18], while in Uttaranchal, India, FDCs accounted for 59% of medicines prescribed [29].

Among the 91 FDCs encountered in the THC center, the most prescribed medicines were vitamins, minerals and dietary supplements, accounting for 40.6% of total FDCs, followed by analgesic/antipyretics at 26.3%. A study by Sarkar et al. found that the most widely prescribed FDCs without a rational basis were multivitamin combinations (31.3%) and cough and cold remedies (17.1%) [28]. A similar study in Duwakot health care center, Kathmandu, found that the antipyretics, antibiotics and NSAIDs to be the most commonly prescribed medicines [30]. Among the 91 FDCs, 50.5% cost less than $1.25, followed by 29.6% medicines in the range of $1.2 - $ 2.5. The finding was similar to a study in a teaching hospital in Pokhara, Western Nepal, where the average cost of medicines per prescription was $2 [31].

Many medicines encountered in the study were based on established regulatory guidelines such as the national model list of essential medicines for Nepal, the WHO Model List of Essential Medicines, and the NNF. In the PHC center, however, the scenario was different than the THC center. Out of the total 91 FDCs prescribed, only 9.8% were from the EDL of Nepal, 12% were from the NNF, and 15.3% were from the WHO Model List of Essential Medicines. This reflects the ignorance of more qualified physicians at secondary and tertiary levels, towards EDL, NNF and WHO model drug list whereas prescribers at primary health care level has better access and information about them. Our findings are supported by a similar study in a tertiary care hospital in Nepal where only 0.8% and 2.1% of FDCs were in accordance with the Nepal and WHO recommended lists of FDCs respectively [28]. However, a report by Shewade et al. had documented that almost 61.3% of the FDCs prescribed in Pondicherry, India, were in accordance with those listed in the WHO recommended list of FDCs [32]. Although a low usage of FDCs from EDLs does not always suggest the inappropriate use, the low prevalence of prescribed FDCs from the established guidelines and formularies in this study (despite being available free or at low cost by governments), indicate that their use was poor. This lead to the irrational use of other FDCs that has no any established therapeutic justification.

### Study limitations

The present study had few limitations. The study was carried out only for a quarter of a year, from February 3rd to May 3rd 2009, and hence seasonal variation in disease prevalence and prescribing patterns might have not been noticed. Background of prescribers at primary, secondary and tertiary health care centers was not studied, as their educational background could be potential confounders in their prescribing practices. In addition, only 100 prescriptions from each setting (primary 100, secondary 100 and tertiary 100) were included for analysis, which may not represent the total population attending each health care centers. Also, this study was conducted few years back in 2009; the current trends on medication use, their prevalence and availability might differ now as several interventions might have been initiated to minimize the use of FDCs.

### Recommendations

Educational interventions to improve prescribing practices at different levels may be required. There is a need to strengthen the mechanism for continuing professional development of practitioners to ensure that they possess the necessary skills and knowledge to prescribe rationally. Government agencies and non- government health organizations should take a lead in this initiative. The medical and pharmacy schools must take the responsibility to train their students and young practitioners the way to access new combinations more logically and should be based on evidence. There is also need for adequate awareness program for the consumers to be aware of the hazards of irrational FDCs with careful monitoring and censoring the misleading claims by pharmaceutical industries. Further studies over a long period of time are required to provide a baseline data of utilization of FDCs which will be beneficial for future longitudinal studies. Extensive studies on comparison between the private and government healthcare centers are urgently required.

## Conclusions

Study revealed FDCs are being extensively utilized among all three levels of health care centers both in private and public sectors. Adherence to established guidelines and EDLs was much lower in private centers than in the public ones and so was the case of cost of FDC prescribing which showed a higher cost among private sectors. The use of essential medicines was low, and the FDCs prescribing were not warranted in most occasions. A needless use of excessive vitamins, minerals and dietary supplements, antimicrobials and analgesic/antipyretic FDCs was noticed in private sectors. Education and awareness to improve prescription practices at different healthcare levels may be deemed necessary.

## Additional file

Additional file 1: International Network for Rational Use of Drug (INRUD) encounter form (modified). (DOCX 13 kb)

## Abbreviations

ADR: Adverse Drug Reaction
EDL: Essential Drug List
FDC: Fixed Dose Drug Combination
HP: Health Post
INRUD: International Network for Rational Use of Drug
IQR: Interquartile Range
MTH: Manipal Teaching Hospital
NNF: Nepalese National Formulary
NPRs: Nepalese Price Rupees
NSAIDs: Nonsteroidal Anti-inflammatory Drugs
PHC: Primary Health Care
SHC: Secondary Health Care
SPSS: Statistical Package for the Social Sciences
THC: Tertiary Health Care
VDC: Village Development Committee
WHO: World Health Organization
