# Supplementary material for: Assessment of utilization pattern of fixed dose drug combinations in primary, secondary and tertiary healthcare centers in Nepal: a cross-sectional study
Source: BMC Pharmacol Toxicol. 2017 Nov 2;18:69. doi: 10.1186/s40360-017-0176-z (PMC5667497; doi:10.1186/s40360-017-0176-z)
Supplement: Additional file 1: — International Network for Rational Use of Drug (INRUD) encounter form (modified). (DOCX 13 kb) [file 40360_2017_176_MOESM1_ESM.docx]

| **ENCOUNTER FORM** | | | | | | | | | | | |  |
| --- | --- | --- | --- | --- | --- | --- | --- | --- | --- | --- | --- | --- |
| Hospital No | Patient identifier (Name) | Age | | Sex | | Prescriber | | | Date | | |  |
|  |  |  | |  | |  | | |  | | |  |
| Health problem | Health problem description/diagnosis | | | | | Department | | | | | |  |
|  |  | | | | |  | | | | | |  |
|  |  |  |  |  |  |  |  |  |  |  |  |  |
| Drugs | Prescription character | | | | | Label | | | | Patient Drug Knowledge | |  |
|  |  |  |  |  |  |  |  |  |  |  |  |  |
|  | Drugs name, strength and dose | | Dispensed Quantity  (DQ) | | Cost per DQ | Patient | Drug | When | | When | How | |
|  |  |  |  |  |  | Name | Name |  |  |  | Much | |
|  |  |  |  |  |  | (0/1) | (0/1) | (0/1) | | (0/1) | (0/1) | |
|  |  | |  | |  |  |  |  | |  |  | |
|  |  | |  | |  |  |  |  | |  |  | |
|  |  | |  | |  |  |  |  | |  |  | |
|  |  | |  | |  |  |  |  | |  |  | |
|  |  | |  | |  |  |  |  | |  |  | |
|  |  | |  | |  |  |  |  | |  |  | |
|  |  | |  | |  |  |  |  | |  |  | |
|  |  | |  | |  |  |  |  | |  |  | |
|  |  | |  | |  |  |  |  | |  |  | |
|  |  | |  | |  |  |  |  | |  |  | |

**Appendix 1**

**International Network for Rational Use of Drug (INRUD) encounter form (modified)**
